# A KL Divergence-Based Loss for In Vivo Ultrafast Ultrasound Image Enhancement with Deep Learning

**DOI:** 10.3390/jimaging9120256

**Published:** 2023-11-23

**Authors:** Roser Viñals, Jean-Philippe Thiran

**Affiliations:** 1 Signal Processing Laboratory 5 (LTS5), École Polytechnique Fédérale de Lausanne (EPFL), 1015 Lausanne, Switzerland; jean-philippe.thiran@epfl.ch; 2 Department of Radiology, University Hospital Center (CHUV) and University of Lausanne (UNIL), 1011 Lausanne, Switzerland

**Keywords:** deep learning, image reconstruction, quality enhancement, ultrafast ultrasound imaging

## Abstract

Ultrafast ultrasound imaging, characterized by high frame rates, generates low-quality images. Convolutional neural networks (CNNs) have demonstrated great potential to enhance image quality without compromising the frame rate. However, CNNs have been mostly trained on simulated or phantom images, leading to suboptimal performance on in vivo images. In this study, we present a method to enhance the quality of single plane wave (PW) acquisitions using a CNN trained on in vivo images. Our contribution is twofold. Firstly, we introduce a training loss function that accounts for the high dynamic range of the radio frequency data and uses the Kullback–Leibler divergence to preserve the probability distributions of the echogenicity values. Secondly, we conduct an extensive performance analysis on a large new in vivo dataset of 20,000 images, comparing the predicted images to the target images resulting from the coherent compounding of 87 PWs. Applying a volunteer-based dataset split, the peak signal-to-noise ratio and structural similarity index measure increase, respectively, from 16.466 ± 0.801 dB and 0.105 ± 0.060, calculated between the single PW and target images, to 20.292 ± 0.307 dB and 0.272 ± 0.040, between predicted and target images. Our results demonstrate significant improvements in image quality, effectively reducing artifacts.

## 1. Introduction

Ultrasound (US) imaging is widely used in medical imaging due to its real-time ability to produce high-quality images of soft tissues. In particular, a technique achieving frame rates of multiple kilohertz called ultrafast US has revolutionized US imaging. The high frame rates achieved by ultrafast US can be exploited to study fast changes in the human body and have enabled new imaging modalities such as shear-wave elastography, which analyzes the tissues’ viscoelasticity, or ultrafast Doppler imaging for flow imaging [1].

Traditional US uses focused beams to scan the imaging plane line by line, whereas ultrafast US transmits a single unfocused wavefront such as a diverging wave (DW) or a plane wave (PW) [1]. While focused beams concentrate energy in narrow beams, unfocused wavefronts disperse energy across the entire field of view. Consequently, imaging with unfocused beams yields lower-amplitude backscattered echoes and a lower signal-to-noise ratio (SNR), resulting in lower contrast. Contrast is also degraded by artifacts caused by grating lobes (GLs) and side lobes (SLs). Furthermore, ultrafast acquisitions suffer from lower lateral resolution due to broader main lobes of the point spread function, compared to line by line acquisitions.

A technique to improve the image quality of ultrafast US images is coherent plane wave compounding (CPWC). This strategy coherently compounds multiple images obtained from unfocused wavefronts steered at different angles. Therefore, this technique suffers from a trade-off between image quality, which is enhanced by increasing the number of compounded acquisitions, and frame rate, which is reduced [2]. Furthermore, coherent compounding assumes that, during acquisition, the region of interest is stationary. Consequently, images acquired on fast-moving areas might suffer from severe motion artifacts.

Several deep-learning-based techniques have been proposed to enhance the image quality of ultrafast acquisitions [3,4,5,6,7,8,9,10,11,12,13]. These approaches are intended to reduce the artifacts caused by GLs and SLs while preserving the speckle patterns, as they comprise positional information of the underlying physical phenomena. While [3,4,5,6,7,8] focus on enhancing the image quality of single PW acquisitions, others intend to improve the quality of the compounding of few PWs [9,10,11] or DWs [12,13].

Most of these studies use convolutional neural networks (CNNs) that learn the mapping between an input image, acquired with one or a few unfocused acquisitions, and a target image resulting from the compounding of several unfocused acquisitions [3,4,8,9,11,12,13]. Perdios et al. [3,4] trained a U-Net-based network [14], using radio frequency (RF) images acquired with single PWs as input and RF images resulting from synthetic aperture (SA) acquisitions as the target. In a related work by Lu et al. [8], the authors proposed a CNN inspired by U-Net [14] and GoogLeNet [15]. Their objective was to enhance the beamforming of single unfocused acquisitions by training a CNN with RF images corresponding to unsteered single PW acquisitions as input and in-phase and quadrature (IQ) data resulting from compounding with three and five PWs as target. Gasse et al. [9] improved the contrast ratio and lateral resolution of RF images resulting from the compounding of three PWs using a CNN with four hidden layers, where the images resulting from the compounding of 31 PWs served as target images. Jansen et al. [11] presented a deep-learning-based reconstruction method in the Radon domain using a U-Net [14]. Their approach successfully enhanced the image quality of images acquired with three PWs, using the compounding of 51 PWs as target images. Finally, Lu et al. [12,13] trained a CNN with five hidden layers using beamformed images acquired with three DWs tilted at different angles as input images and the images formed by compounding 31 DWs as target images. In [12], RF images were used, while in [13], IQ images were considered.

Using focused acquisitions as target images has also been proposed [5,10]. Zhou et al. [5] employed a generative adversarial network (GAN) with RF images acquired with one PW as input. Khan et al. [10] implemented a CycleGAN to enhance the B-mode image quality resulting from different numbers of compounded acquisitions: 3, 7, 11, and 31 PWs. Alternatively, recent studies have explored the use of self-supervised learning with a 12-layer network to enhance the quality of single unfocused acquisitions without the need of target images [6,7].

The proposed methods have been trained using different types of data, including simulated data [3,4,6,7,8], a combination of in vivo and in vitro (phantom) images [7,9,10,12,13], or exclusively in vitro acquisitions [11]. However, some models are evaluated on acquisitions taken on the same phantoms used for training, hindering the assessment of their generalization capabilities [9,10,11,12,13]. Furthermore, existing models are typically tested on a limited amount of in vivo data, predominantly with acquisitions from the carotid/thyroid region [3,4,7,8,10], or even without considering in vivo acquisitions [6,11].

Acquiring large human in vivo datasets to develop deep learning methods for ultrafast image improvement is a time-consuming and ethically regulated process. Consequently, there are only a few datasets that contain exclusively in vivo data, such as the one in [5]. Their method was trained, tested, and evaluated using only 360 pairs of RF data acquired on different body parts of 30 healthy volunteers, randomly selecting 36 images for the testing set.

The absence of comprehensive testing across different body regions and large datasets underscores a significant gap in current research. To assess the generalizability of methods, it is crucial to train and test them while excluding similar regions or images from the same volunteer in both training and testing phases. Furthermore, achieving model robustness requires training on large and diverse in vivo datasets to minimize the domain gap between training datasets and in vivo testing data. This work aims to address these limitations and provide a more robust and generalized solution for enhancing the quality of in vivo single unfocused acquisitions.

The authors in [4] proposed a CNN-based US image reconstruction method that not only reduces artifacts and restores the speckle patterns of single ultrafast acquisitions but also can be used for displacement estimation [16]. Although this approach showed potential for recovering high-quality images from single unfocused acquisitions using simulated data, the quality improvement dropped significantly when applied to in vivo data due to the domain shift between in vivo and simulated data [4]. Furthermore, their assessment of the method on in vivo acquisitions was limited, lacking quantitative results and only involving a small number of in vivo acquisitions.

The objective of this work is to improve the performance of this approach on in vivo data, by reducing noise and artifacts from single in vivo PW acquisitions to achieve an image quality comparable to that of CPWC with 87 PWs. To accomplish this, we have improved the previous method [4] by slightly modifying the CNN architecture, proposing a new training loss function, and training and assessing it with a large and new in vivo dataset.

This work introduces two significant contributions that aim to improve the ultrafast US image quality of RF images:

1. A novel loss function that effectively handles the high dynamic range of the RF images while preserving the probability distribution function of the echogenicity values.
2. A large and diverse in vivo dataset comprising 20,000 images. This dataset has been used for training the CNNs and will be made available for public access along with this paper.

The remaining sections of the paper are structured as follows. In Section 2, we provide a detailed overview of the materials and methods employed in this study. This section is organized into five subsections, covering dataset acquisition and preprocessing, the architecture and training of the CNN, the dataset splitting strategies, the training losses, and the performance evaluation and metrics. Moving on to Section 3, we present the results, which are divided into two distinct subsections: one that compares two different training loss functions, and another that delves into the network’s ability to generalize across various body regions. Section 4 is divided in five subsections that include an in-depth discussion of the results, a comparison with state-of-the-art methods, an analysis of the computational efficiency of our method, and limitations and future work. Finally, in Section 5, we summarize the main conclusions drawn from this study.

## 2. Material and Methods

### 2.1. Dataset Acquisition and Preprocessing

A large dataset of 20,000 in vivo images acquired on different body parts was collected from nine healthy volunteers (five males and four females), with ages ranging from 22 to 33 years, as outlined in Table 1. Between two consecutive acquisitions, a brief pause of a few seconds was allowed, during which the probe was repositioned to a new location. The acquisitions were performed with the approval of the Cantonal Commission on Ethics in Human Research (2022-01696, CER-VD, Vaud, Switzerland). An in vitro image was also acquired on the CIRS model 054GS phantom (CIRS, Norfolk, VA, USA) to assess the performance of our method and derive normalization matrices. The acquisitions were collected using the GE 9L-D linear array transducer (GE Healthcare, Chicago, IL, USA), a linear array transducer with 192 elements and a center frequency of 5.3 MHz, and the Vantage 256 system (Verasonics, Kirkland, WA, USA).

Each acquisition consisted of 87 PWs steered at different angles acquired at a pulse repetition frequency of 9 kHz. An alternating steering angle sequence [17] with a steering angle spacing of 0.38${}^{\circ }$ was employed, resulting in the steering angles of the PWs being evenly spaced between −16.34${}^{\circ }$ and 16.34${}^{\circ }$. The steering angle spacing and the number of steered acquisitions were determined such that the focusing quality was comparable to that of the optimal multi-focus, as described in [2,4], considering an F-number of 1.75. Time gain compensation was applied assuming a tissue attenuation of 0.5 dB/(cm·MHz).

The ultrasound probe was moved before each measurement to ensure that each acquisition was distinct from the previous one. The maximum frame rate between two acquisitions was restricted to 47.5 Hz, maintaining an intensity spatial peak temporal average (ISPTA) below the Food Drug Administration (FDA) recommended threshold of 94 mW/cm^2^ [18]. The peak-to-peak voltage was set to 40 V to ensure a mechanical index below 0.7, as recommended by the British Medical Ultrasound Society (BMUS) [19]. The imaging configuration and parameters used are specified in Table 2.

Ultrafast US imaging can be formulated as an inverse problem [20]. Let us consider the measurements y$\in R^{N}$, the measurement noise ϵ$\in R^{N}$, the measurement model operator H$:R^{M}\to R^{N}$, and the vectorized image that we want to estimate θ $\in R^{M}$. Then, the inverse problem can be formulated as finding θ such that y=Hθ+ϵ.

Our reconstruction pipeline relies on the estimation of a solution to this inverse problem. This estimation was obtained following the method described in [4] with a backprojection-based delay-and-sum operator that was implemented using PyUS [21], a GPU-accelerated Python package for US imaging. A λ/8×λ/8 grid with a width spanning the probe aperture and a depth from 1 mm to 55 mm was considered, resulting in images of 1483 × 1189 pixels.

From each acquisition, we estimated two beamformed RF images. The first corresponds to the single unfocused acquisition obtained from the PW measurement steered at 0${}^{\circ }$, and it is referred to as the input image. The second results from coherently compounding the 87 PWs acquisitions steered at different angles and is referred to as the target or CPWC image.

Using 1000 speckle image pairs acquired on the CIRS model 054G phantom, we computed two normalization matrices: one for the input and the other for the target images. These matrices are designed to compensate for the variations in echogenicity introduced by beamforming, ensuring that B-mode images of the in vitro phantom with uniform echogenicity appear consistently uniform with 0 dB echogenicity. We first beamformed the speckle images. Afterward, we detected the envelope and log-compressed the resulting images to generate the B-mode images. These B-mode speckle images were averaged, giving rise to a matrix of 1483 × 1189 values. By converting the B-mode average matrices to linear scale, we obtained the normalization matrices. These normalization matrices were applied to normalize all the RF images by dividing the RF images by them. The vectorized normalized RF image corresponding to the single unfocused acquisition is denoted as $x_{1\mathrm{PW}}$ $\in \;R^{M}$, while the one corresponding to the target image is denoted as $x\in R^{M}$.

To evaluate the diversity of our datasets, the probability distributions of the B-mode values of the normalized images, $x_{1\mathrm{PW}}$ and x, were analyzed. The mean and standard deviation of these distributions for both imaging modalities are presented in Table 1. We observe that our images span a high dynamic range, which significantly varies across different imaged body areas. Furthermore, the single unfocused images tend to have higher echogenicity and a narrower range compared to the target images, leading to reduced contrast. The lower echogenicity in the target images compared to input images is primarily due to two factors. First, motion can occur between PW acquisitions, reducing coherence between acquired signals. Second, the effects of multiple scattering are supposed to be incoherent between different insonifications and therefore are reduced by coherent compounding.

### 2.2. CNN Architecture and Training

**Figure 1:**
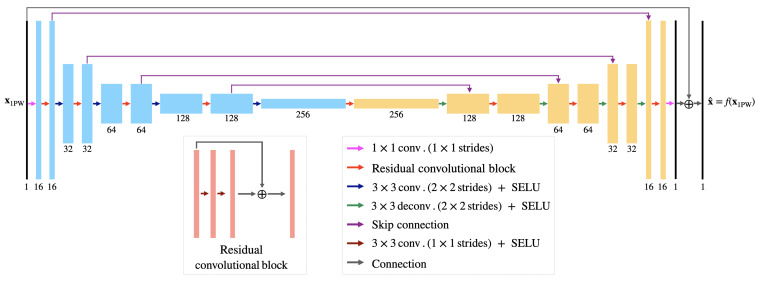
Convolutional neural network (CNN) architecture and the residual convolutional blocks considered. Arrows represent network layers and operations, while rectangles represent tensors with the number of channels specified below them.

Our CNN architecture is based on the U-Net architecture described in [4]. It has previously demonstrated success in enhancing ultrafast ultrasound images by effectively mitigating artifacts from GLs and SLs when trained on simulated data. The network architecture, illustrated in Figure 1, consists of multiple multichannel convolutional layers and scaled exponential linear unit (SELUs) organized into downsampling and upsampling paths. The main modification from the architecture presented in [4] is the replacement of the rectified linear unit activation functions with the SELU activation functions [22]. Notably, with this activation function, we observed that our network converged faster in our specific setup, showing improved training efficiency.

The initial layer of the network (pink arrow) expands the input image’s channels to 16. It is followed by the downsampling path, which concatenates a series of residual convolutional blocks (red arrows) and downsampling layers (blue arrows), which simultaneously increase the number of channel and reduce the spatial dimensions. The upsampling path consists of a sequence of skip connections (violet arrows), residual convolutional blocks (red arrows), and upsampling layers (green arrows). In the end, the number of channels in the output is reduced to match those of the initial input image (pink arrow), and the output is summed with the input image (gray arrow).

The network aims to learn the mapping $f:R^{M}\to R^{M}$ between $x_{1\mathrm{PW}}$ and x in order to estimate higher-quality images, $\hat{x}$, from the PWs steered at 0${}^{\circ }$: $\hat{x}=f\left(x_{1\mathrm{PW}}\right)$. Thus, the CNN was trained using as input images the estimated normalized RF images corresponding to the PWs steered at $0^{\circ }$, $x_{1\mathrm{PW}}$, and as target images the estimated normalized RF images resulting from the 87 PWs compounded acquisitions, x. The choice of training the CNN with RF images is driven by the need for better-quality ultrafast ultrasound images, essential for achieving more accurate speckle tracking.

The training pipeline was implemented using PyTorch (v1.12), and the trainings were executed on an Nvidia Tesla V100 GPU (Nvidia Corporation, Santa Clara, CA, USA). The network was trained for 20 epochs using 16 channels and an Adam optimizer [23] with a learning rate of 0.0003 and a weight decay of 0.005. The training batch size was set to 16 and a random shuffle was applied on every epoch. All these parameters’ values were optimized using Optuna [24], a software that implements a Bayesian optimization algorithm for hyperparameter tuning.

### 2.3. Dataset Splitting Strategies

Two different dataset splitting strategies were considered. Firstly, to prevent the inclusion of similar images from the same volunteer in both the training and validation or test sets, we performed a volunteer-based split.

Secondly, to assess the network’s generalizability across different body regions and the dataset diversity, we adopted a splitting strategy that we name region-based splitting. With this approach, all images in the dataset that do not belong to a specific body region were randomly divided between the training set (90%) and the validation set (10%). The testing set exclusively contains the image pairs acquired from the specific body region.

With these two strategies, we define three different splits:

- **Volunteer-based split (VS)**: The dataset is split using the volunteer-based strategy. Out of the 9 volunteers, 6 have been used for training, 1 for validation, and 2 for testing.
- **Carotid split (CS)**: The dataset is split using the region-based strategy, with the test set including all image pairs acquired on the carotids from all the volunteers. The testing set of the CS will be referred to as the carotid test set.
- **Back split (BS)**: The dataset is split using the region-based strategy, with the test set including all image pairs acquired on the back from all the volunteers. The testing set of the BS will be referred to as the back test set.

From the VS test set, we derive two additional test subsets: one consisting of images acquired on the carotids of the two test volunteers and the other comprising images acquired on the backs of the two test volunteers. We refer to these two test subsets as the VS carotid test subset and the VS back test subset. It is important to note that all images included in these two sets are also part of the carotid test set and the back test set, respectively.

The resulting number of images of the three different splits are detailed in Table 3.

### 2.4. Training Losses

Due to the high dynamic range of our RF images, traditional losses such as mean absolute error and mean squared error are not suitable. To address this issue, the authors in [4] introduced a log-compressed loss named mean signed logarithmic absolute error (MSLAE) that showed a great potential to train networks with RF simulated images of high dynamic range. This loss can be expressed as follows:

(1) $$L_{\mathrm{MSLAE}}\left(x,\hat{x}\right)=\frac{1}{n}{\left|\left|g_{\alpha }\left(x\right)-g_{\alpha }\left(\hat{x}\right)\right|\right|}_{1},$$

with

(2) $$g_{\alpha }\left(x_{m}\right)=\mathrm{sign}\left(x_{m}\right)\log_{\alpha }\left(\frac{\alpha }{\max(\alpha ,\left|x_{m}\right|)}\right)$$

where $x_{m}$ denotes the pixel *m* of the vectorized image x and α∈(0,1).

When using this loss with our in vivo dataset, the network tends to widen the echogenicities distribution and shift them to lower echogenicities.

A well-known measure to quantify the similarity between two probability distributions is the Kullback–Leibler (KL) divergence. It is a non-symmetric measure of the difference between two distributions. Let us consider two probability distributions $p\left(z\right):R^{M}\to R^{K}$ and $q\left(\hat{z}\right):R^{M}\to R^{K}$, with *M* and *K* denoting the number of samples and bins, respectively. Then, the KL divergence of $q(\hat{z})$ from p(z) is defined as

(3) $$D_{KL}\left(p\left(z\right)\left|\right|q\left(\hat{z}\right)\right)=\sum_{k=0}^{K}p{\left(z\right)}_{k}\ln\frac{p{\left(z\right)}_{k}}{q{\left(\hat{z}\right)}_{k}},$$

where $p{\left(z\right)}_{k}$ and $q{\left(\hat{z}\right)}_{k}$ are the probability estimates of the *k*-th bin. To improve the performance of the image enhancement method, we introduce a new loss named KLD-MSLAE that aims to reduce diffraction artifacts while preserving the echogenicity distribution probabilities by combining MSLAE with the KL divergence. It is defined as follows:

(4) $$\begin{array}{c} L_{\mathrm{KLD}-\mathrm{MSLAE}}\left(x,\hat{x}\right)=L_{\mathrm{MSLAE}}\left(x,\hat{x}\right)\\ +\beta D_{KL}\left(p\left(z\right)\left|\right|q\left(\hat{z}\right)\right), \end{array}$$

where β∈R is a weighting factor, and p(z) and $q(\hat{z})$ denote the estimated probability distributions of $z=20\log_{10}\left(\max\left(\alpha ,\left|x\right|\right)\right)$ and $\hat{z}=20\log_{10}\left(\max\left(\alpha ,\left|\hat{x}\right|\right)\right)$, respectively.

The probability distributions p(z) and $q(\hat{z})$ have to be estimated so that the estimates are differentiable. We consider that our probability distributions span over the range $\left[-\gamma_{\mathrm{dB}},\gamma_{\mathrm{dB}}\right]$, and we set the number of bins to *K*. Each bin *k* has a width of $\delta =2\gamma_{\mathrm{dB}}/K$ and is centered at $c_{k}=-\gamma_{\mathrm{dB}}+\left(k+0.5\right)\delta$, with k=0,…,K. Then, we can define $\Delta_{m,k}=z_{m}-c_{k}$. The probability distribution on the *k*-th bin, $p{\left(z\right)}_{k}$, can be approximated by

(5) $$p{\left(z\right)}_{k}=\frac{\sum_{m=1}^{M}\left(s_{\eta }\left(\Delta_{m,k}+\frac{\delta }{2}\right)-s_{\eta }\left(\Delta_{m,k}-\frac{\delta }{2}\right)\right)}{\sum_{k=1}^{K}\sum_{m=1}^{M}\left(s_{\eta }\left(\Delta_{m,k}+\frac{\delta }{2}\right)-s_{\eta }\left(\Delta_{m,k}-\frac{\delta }{2}\right)\right)},$$

with $s_{\eta }\left(x\right)=1/\left(1+e^{-\eta x}\right)$ denoting the logistic function with a growth rate of η.

The probability distribution estimation depends on the choice of three parameters: η, *K*, and $\gamma_{\mathrm{dB}}$. A larger number of bins *K* and a steeper logistic function enhance the accuracy of the estimation. Our parameter choices were made as follows. Firstly, as we increase η, the logistic function will approach a Heaviside step function, becoming less differentiable. We opted for a logistic growth η of 0.5, as increasing it further resulted in training instabilities. Secondly, to speed up the computation of the KL divergence term, we employed a matrix-based implementation for our probability distribution estimations. Unfortunately, due to memory constraints, we had to limit the number of bins *K* to 40. Thirdly, to mitigate the widening and shifting echogenicity effects observed during training with the MSLAE loss, we needed to consider a wide range of echogenicity values, which we controlled with the parameter $\gamma_{\mathrm{dB}}$. After training with various values, we found that $\gamma_{\mathrm{dB}}=60$ dB provided the best results.

In both components of the loss, the parameter α plays a key role. For any RF value *x* satisfying |x|<α, the $g_{\alpha }\left(x\right)$ of the MSLAE term is equal to zero while the KL divergence term ignores it. Therefore, the α value prevents the network to learn from absolute RF values lower than α or, equivalently, from echogenicities lower than $\alpha_{\mathrm{dB}}=20\log_{10}\left(\alpha \right)$. Furthermore, it sets a threshold that allows the use of logarithmic operations in the losses without facing the vertical asymptote of the logarithmic function in 0. Different α values were used to train the network. By visually assessing the resulting images, we observed that the best results are obtained with $\alpha_{\mathrm{dB}}=-60$ dB. Note that this low value of $\alpha_{\mathrm{dB}}$ does not restrict the network’s ability to learn from low echogenicities present in the dataset. It is important to emphasize that $\gamma_{\mathrm{dB}}$ and $\alpha_{\mathrm{dB}}$ have been optimized specifically for the echogenicity distribution of our dataset. The selection of $\alpha_{\mathrm{dB}}$ ensures that the network learns from the lower echogenicity values of the input distribution, while $\gamma_{\mathrm{dB}}$ is chosen to calculate the KL divergence term of the loss, taking into account the entirety of echogenicity ranges present in both the input and target distributions. Therefore, these two values should be modified accordingly when considering other data distributions.

Finally, another parameter demanding tuning is the weight parameter, denoted as β. When β assumes a low value, the echogenicity distributions tend to become broader and shifted, similar to when training exclusively with the MSLAE loss. Conversely, a high β value leads to improved echogenicity in the results but can limit the network’s ability to remove artifacts effectively, potentially introducing a blurry effect in the resulting images. We conducted several trainings with varying β values and, after quantitative and qualitative assessment, we ultimately set β=0.5 as it achieved a favorable balance between artifact removal and desirable echogenicity distributions. When working with other data distributions, it is necessary to adjust the parameter β because it controls the distribution shift performed by the network when trained with MSLAE, and this shift varies depending on the input distribution.

### 2.5. Performance Evaluation and Metrics

To evaluate the performance of our method, we compare the outputs of the CNN to the corresponding target test images acquired with 87 PWs, which we regard as ground truth. Three metrics are considered: the structural similarity index measure (SSIM) [25], the peak signal-to-noise ratio (PSNR) [25], and the KL divergence (Equation (3)). These metrics are computed between the B-mode images within the range of [−40 dB, 40 dB], even though the trainings were performed on RF beamformed images. Furthermore, we calculate the means and standard deviations of the resulting echogenicity values.

The contrast (C) is assessed in selected areas of two test images. The contrast between two image areas is calculated on the envelope-detected images following [4]. Specifically, the contrast between two designated areas, denoted as *A* and *B*, is computed in decibels as $C=20\cdot \log_{10}\left(\bar{s_{A}}/\bar{s_{B}}\right)$. Here, $\bar{s_{A}}$ and $\bar{s_{B}}$ represent the mean values of the envelope-detected images in regions *A* and *B*, respectively.

For the assessment of speckle patterns, the SNR is calculated in selected areas of the same two test images. The SNR is computed as the ratio of the mean value to the standard deviation: $\mathrm{SNR}=\bar{s_{A}}/\sigma_{s_{A}}$, where $\bar{s_{A}}$ and $\sigma_{s_{A}}$ denote the mean and standard deviation of the amplitude of the envelope-detected image in the region *A*, respectively. For an ideal Rayleigh distribution, the expected SNR is 1.91 [4].To further evaluate speckle patterns and their resolution, the axial and lateral full width at half maximum (FWHM) of the axial and lateral dimensions of the 2D autocovariance function (ACF) [4] is computed within the same areas containing the speckle patterns.

Our reconstruction method is also evaluated on an in vitro image taken on the CIRS model 054GS phantom. This image contains three inclusions with different contrasts: one anechoic inclusion and two low-echogenic inclusions with a C of −6 dB and −3 dB, respectively. All three inclusions are located at a depth of 40 mm and have a diameter of 8 mm. As with the two in vivo images, we compute the contrasts of these inclusions. We also evaluate the speckle patterns by computing the SNR and the FWHM of the 2D ACF. This assessment is performed within selected areas exclusively containing speckle patterns.

## 3. Results

### 3.1. Comparison of KLD-MSLAE and MSLAE Losses

**Figure 2:**
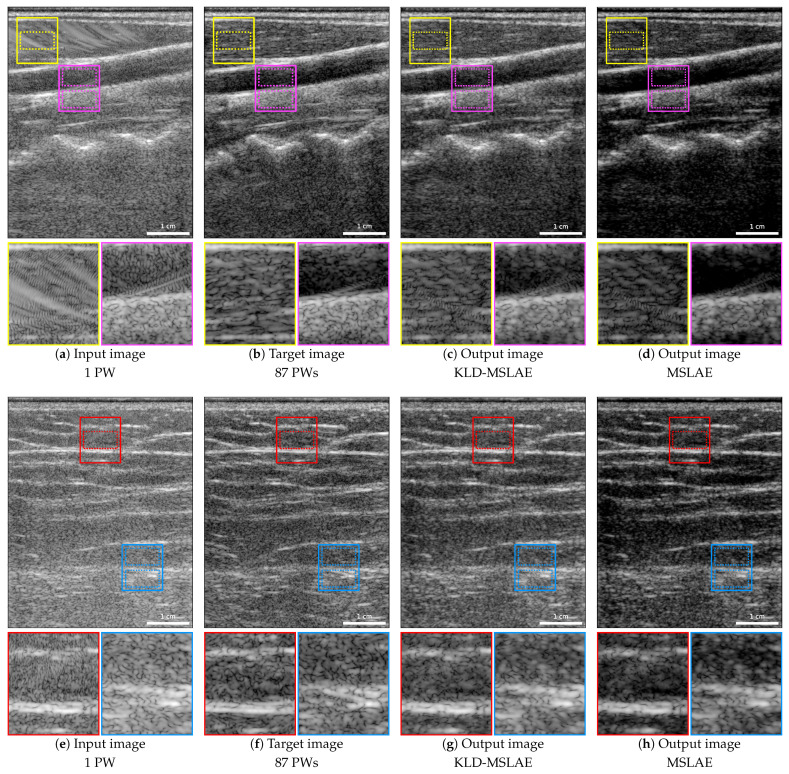
B-mode images with a dynamic range of 65 dB (−25 to 40 dB) of the carotid (**top row**) and back (**bottom row**) of two test volunteers: (**a**,**e**) input images acquired with one PW; (**b**,**f**) target images obtained from the coherent compounding with 87 PWs; (**c**,**g**) resulting images from the CNN trained with the KLD-MSLAE loss; (**d**,**h**) resulting images from the CNN trained with the MSLAE loss.

To assess the improvement achieved with the KLD-MSLAE loss compared to the MSLAE loss, we trained our CNN using both loss functions, applying the VS. Figure 2 shows the input, target, and output images of two acquisitions. The first row shows a carotid artery of one of the volunteers of the test set, while the second row shows an acquisition taken on the back of the other test volunteer.

The improvement in terms of the reduction of artifacts is noticeable using both losses. Particularly, this improvement can be clearly observed in the area outlined in yellow in the carotid images, where a large artifact is highly visible in the input image (Figure 2a), and the area delimited in red in the back image (Figure 2e). When zooming in on both areas, we can observe that the artifacts have been reduced and that some speckle patterns hidden or modified by the artifacts have been restored. To evaluate the restoration of speckle patterns, the SNR and the axial and lateral FWHM of the 2D ACF were computed in the areas delimited by yellow and red dotted lines. The resulting values are specified in Table 4.

It is important to acknowledge that the target images might also be affected by artifacts, such as the SLs present in the region highlighted in magenta (Figure 2b). These SLs are partially attenuated but not entirely removed by the CNN, as shown in the magenta areas of Figure 2c,d.

When using the MSLAE loss, the images exhibit increased contrast. Particularly, there is an over-attenuation of the low-echogenic areas, which is evident in the deeper area of Figure 2d. In contrast, the KLD-MSLAE loss attains a comparable contrast to the target images. To quantify this, the contrasts between the upper and lower areas delimited in magenta and blue dotted lines have been computed and are presented in Table 4.

**Figure 3:**
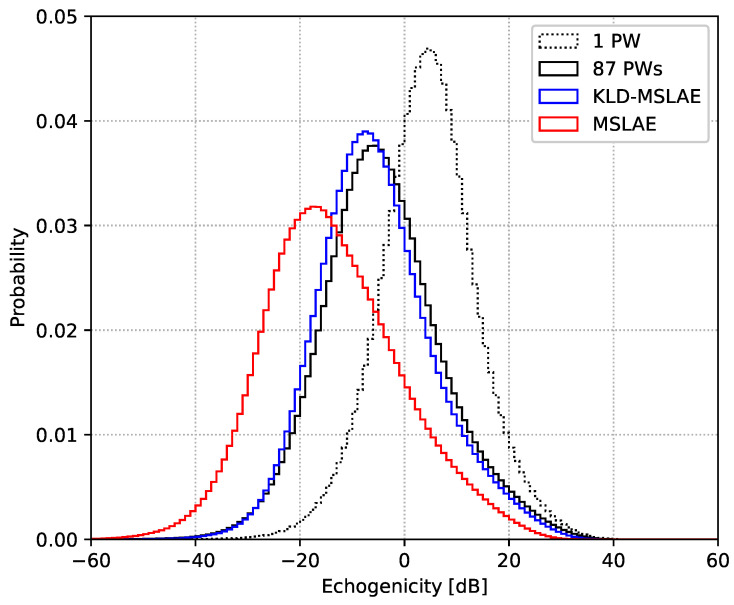
Probability distributions of echogenicity values in the VS test set for input, target, and output images of the CNN. The CNN was trained using both the KLD-MSLAE loss and the standalone MSLAE loss.

To further analyze the discrepancies arising from training with the two different losses, Figure 3 presents the probability distributions of B-mode values for the input, target, and CNN’s output images of the test set. It is evident that the CNN trained with the MSLAE loss causes the echogenicity distribution to widen and shift toward lower values. Conversely, training with the KLD-MSLAE loss enables the CNN to achieve a distribution of echogenicity closer to that of the target images.

The reconstructed B-mode images were compared to the target images using the metrics PSNR, SSIM, and KL divergence. Table 5 presents the mean and standard deviation of these metrics across all test set acquisitions, along with the mean and standard deviation of the resulting echogenicity values. From these results, it is evident that the CNN, when trained with the KLD-MSLAE loss, enhances both the PSNR and SSIM with respect to the target images, in comparison to the CNN trained with the MSLAE loss. Furthermore, the KL divergence between the output and target images is also highly improved. A lower KL divergence indicates a higher similarity in echogenicity distributions and, consequently, a closer resemblance in contrast to the target images. The resemblance in echogenicity distributions can also be observed by analyzing the mean and standard deviation of the resulting echogenicity values. The CNN trained with KLD-MSLAE presents a mean and standard deviation closer to the target echogenicity values. In contrast, when trained with MSLAE, the resulting echogenicity values have a mean shifted towards lower values and a higher standard deviation compared to the target values.

**Figure 4:**
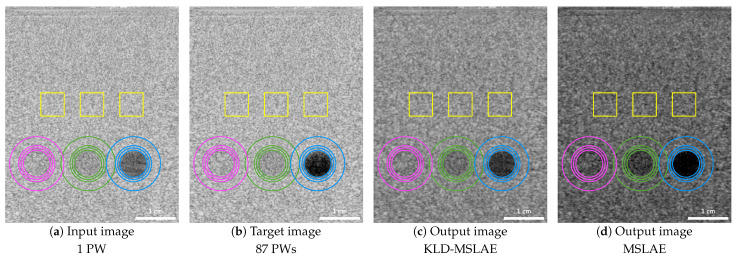
B-mode images with a dynamic range of 65 dB (−45 to 20 dB) of an in vitro acquisition containing two low-echogenic inclusions and an anechoic inclusion: (**a**) input image acquired with one PW; (**b**) target image obtained from the coherent compounding with 87 PWs; (**c**) resulting image from the CNN trained with the KLD-MSLAE loss; (**d**) resulting image from the CNN trained with the MSLAE loss.

The network trained on in vivo data was applied to an in vitro phantom acquisition. Figure 4 shows the input, target, and CNN output images using the two losses. The regions where the contrasts have been calculated are marked with multiple concentric circles. The contrasts are calculated between the inner part of the smaller circles and the background areas between the two outer circles. The two low-echogenic inclusions with a contrast of −3 dB and −6 dB with respect to the background are highlighted in magenta and green, respectively, and the anechoic inclusion is indicated in blue. The speckle patterns are assessed in three regions highlighted in yellow by computing the SNR and the FWHM of the axial and lateral dimensions of the 2D ACF. Table 6 summarizes the resulting metrics.

### 3.2. Network’s Generalizability Across Different Body Regions

To evaluate the network’s ability to generalize across different body regions, we trained our CNN with the KLD-MSLAE loss function using the two region-based dataset splits detailed in Section 2.3.

**Figure 5:**
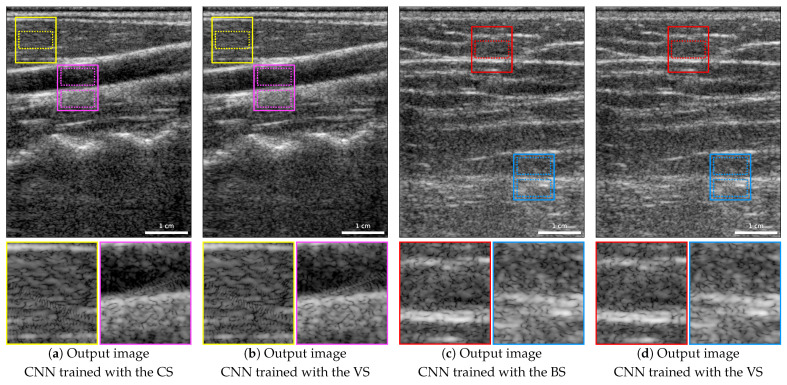
B-mode images with a dynamic range of 65 dB (−25 to 40 dB) of the carotid and back of the two test volunteers: (**a**) resulting carotid image from the CNN trained with the CS; (**b**) resulting carotid image from the CNN trained with the VS; (**c**) resulting back image from the CNN trained with the BS; (**d**) resulting back image from the CNN trained with the VS.

Figure 5 depicts the same carotid artery and back acquisitions as Figure 2. In this figure, we show the resulting image of the carotid artery image when the CNN was trained without any carotid images using the CS (Figure 5a), an image of the back when the CNN was trained excluding back images using the BS (Figure 5c), and, for reference, both images resulting from the CNN trained using the VS (Figure 5b,d). We can observe that the resulting images are visually similar, with significantly reduced artifacts compared to the input images (Figure 2a,e), regardless of whether images from the same region were used for training or not. Specifically, in the zoomed areas highlighted in yellow and red, the artifacts have been considerably reduced and some speckle patterns that were altered or hidden by these artifacts have been restored.

In the regions demarcated by dotted lines within these two areas, the restoration of speckle patterns was assessed by computing the SNR and the axial and lateral FWHM of the 2D ACF. We also measured the contrasts between the upper and lower areas highlighted in magenta and blue dotted lines in the carotid and back images, respectively. Table 7 and Table 8 present the resulting values. Note that, while the resulting images are visually very similar, some differences can be observed on the speckle patterns and contrast metrics. Particularly, the lateral FWHM of the 2D ACF is larger in both images when using the region-based splits instead of the VS, being for the carotid image closer to the target value. Nevertheless, the contrasts are closer to the target ones when training with the two region-based splits.

To analyze the performance across the carotid test set and back test set, we compare the output images from the trainings with the two region-based dataset splits to the target images, using the metrics PSNR, SSIM, and KL divergence. The mean and standard deviation of these metrics across the two test sets, i.e., the test sets of CS and BS, along with the mean and standard deviation of the resulting echogenicity values, are reported in Table 9.

For a fair comparison of the network’s performance when including and excluding body regions in the training, we obtained these same metrics on the VS carotid test subset and the VS back test subset. These two subsets include the images acquired on the carotids and backs of the two volunteers assigned to test in the VS, respectively. We evaluated the performance on the VS carotid test subset by testing two networks: the first trained using the CS and the second with the VS. Similarly, we assessed the performance on the VS back test subset by using again two networks, the first trained with the BS and the second trained using the VS. Table 9 presents the resulting values.

## 4. Discussion

### 4.1. Comparison of KLD-MSLAE and MSLAE Losses

Our deep-learning-based ultrafast ultrasound image enhancement method has proven to successfully reduce artifacts, leading to an improvement in the image quality of single unfocused acquisitions. To compare the two losses, we consider the VS, i.e., different volunteers are used for training, validation, and testing the network. The two in vivo examples demonstrate the CNN’s capability to effectively mitigate artifacts on different body parts. To quantitatively assess the performance, we compute PSNR, SSIM, and KL divergence between the output and target B-mode images and compare them to those between the input and target B-mode images.

By adopting the KLD-MSLAE loss, we achieve an overall enhancement in terms of PSNR and SSIM. Specifically, the PSNR increases from 16.466 ± 0.801 dB to 20.292 ± 0.307 dB, and the SSIM increases from 0.105 ± 0.060 to 0.272 ± 0.040. The KL divergence component of the loss helps to attain a contrast and echogenicity distribution similar to the target images. This fact is evident when comparing the mean and standard deviation of the resulting echogenicity (−5.41 ± 11.25 dB), which is closer to the target echogenicity (−4.18 ± 11.64 dB) than the input (4.48 ± 9.44 dB). Furthermore, the distance between echogenicity distribution can also be assessed using the KL divergence. The KL divergence between input and target echogenicity distributions is 0.303 ± 0.090, which is highly reduced to 0.015 ± 0.015 by the CNN trained with KLD-MSLAE.

In contrast, when training with the MSLAE loss, the achieved PSNR is decreased from 16.466 ± 0.801 to 16.196 ± 1.008 dB, whereas the SSIM is increased from 0.105 ± 0.060 to 0.179 ± 0.036, being both metrics significantly lower those obtained with the KLD-MSLAE loss. The resulting echogenicity of −13.65 ± 13.16 dB is considerably distant from the target of −4.18 ± 11.64 and the KL divergence is 0.258 ± 0.092, being only slightly better than the baseline value of 0.303 ± 0.090.

The MSLAE loss shifts the echogenicity values to lower levels and spans them to a wider range. This induces a higher contrast that results in the loss of fine details and speckle patterns, specially in anechoic regions and greater depths. The fact that MSLAE achieves higher contrast than KLD-MSLAE is further corroborated by analyzing the computed contrasts within the highlighted magenta and blue regions. In both areas, the difference between the contrasts achieved by the CNN trained with the MSLAE loss and the targets are −1.84 and −3.05 dB, respectively. With the KLD-MSLAE loss, these differences with the target values are reduced to 1.74 dB in the magenta area and −0.66 dB in the blue area. Note that both losses yield contrasts closer to the target image contrasts than the single unfocused input images.

Two specific regions, highlighted in yellow and red, that exhibit artifacts that hide or alter the speckle patterns were analyzed. Upon visual assessment, we can observe that the CNN recovers speckle patterns that are more similar to those in the target images when contrasted with the original regions on the single PW images. In the area indicated in yellow of the carotid image, the achieved SNRs when training with KLD-MSLAE and MSLAE are 1.436 and 1.377, respectively, with the latter approaching the target value of 1.261 more closely. The lateral and axial FWHM of the 2D ACF are highly reduced by the CNN, specially when trained with the KLD-MSLAE loss, resulting in values lower than the intended target values. Nevertheless, within the red region of the back image, the FWHM of the ACF in the lateral dimension significantly exceeds the target value of 580.19 μm, being 764.88 μm and 1248.30 μm, with the CNN trained with KLD-MSLAE and MSLAE losses, respectively. Note that the speckle patterns of this specific region of the input image are highly altered by artifacts, leading to an increase in their resolution and rendering them significantly distinct from the speckle patterns in the target image. Despite the increase in the lateral FWHM of the ACF in the red area, the region restored by the CNN is much more similar to the target one than those in the input image. The SNR measured in this region has been improved from 1.134 in the input image to 0.789 in the output image resulting from the CNN trained with KLD-MSLAE, being the target 0.838. It is worth mentioning that, in both regions and in both dimensions, training with the KLD-MSLAE loss results in lower axial and lateral FWHM of the 2D ACF compared to training with MSLAE.

While there is a clear improvement in in vivo data in terms of contrast and artifacts removal, this improvement does not extend to the in vitro phantom image. This disparity could arise from the domain gap between the in vitro data and the training dataset, which comprises vastly different structures and artifacts compared to those present in the in vitro image.

When visually assessing the in vitro image, we can observe that the CNN produces images of lower echogenicity, specially when trained with the MSLAE loss. In the two low-echogenic inclusions with target contrasts of −3.00 dB and −6.27 dB, the contrasts measured in the output images of the CNN trained with KLD-MSLAE are −3.60 dB and −7.38 dB, respectively, whereas when trained with MSLAE are −4.69 dB and −9.13 dB, respectively. Therefore, in both regions and with both losses, the contrasts (in absolute value) are surpassed. Conversely, the resulting contrasts of the anechoic inclusion, −20.31 dB with KLD-MSLAE and −24.04 dB with MSLAE, are lower in absolute value than the target value of −28.35 dB, although representing an enhancement with respect to contrast of −18.33 dB measured in the input image. As observed in the in vivo images, the absolute values of the contrasts in the CNN’s output images, trained with the MSLAE loss, exceed those achieved when trained with the KLD-MSLAE loss. This fact can be attributed to the widening effect observed in the echogenicity distribution when training with MSLAE.

To assess the preservation of speckle patterns, the SNR and the FWHM in both axial and lateral dimensions of the ACF have been computed for three areas containing only speckle patterns. In terms of SNR, when trained with the KLD-MSLAE loss, the CNN slightly improves the SNR, from an SNR in the input image of 1.911 ± 0.024 to 1.895 ± 0.009 in the CNN predicted image, with a target mean SNR of 1.884 ± 0.039. By contrast, training with MSLAE led to a significantly lower SNR of 1.658 ± 0.005 compared to the target. Furthermore, regardless of the loss used, the FWHMs of the ACF, especially in the lateral dimension, exceed the desired values, indicating that the resolution of the speckle patterns in the phantom image is penalized. Notably, the KLD-MSLAE achieves lower FWHM in both dimensions compared to MSLAE, suggesting a better speckle preservation.

### 4.2. Network’s Generalizability Across Different Body Regions

To assess the CNN’s ability to generalize to unknown body regions, the CNN was trained with the CS (excluding carotid images for training) and the BS (excluding back images for training), using the KLD-MSLAE loss. The results were compared to those with VS (including carotid and back images for training) on the VS carotid test subset and the VS back test subset.

Upon visual comparison of two images from these two test subsets, training with CS or BS produces similar results to training with VS. Notably, both carotid and back images exhibit fewer artifacts, specifically visible in the areas outlined in yellow for carotid images and red for back images.

The speckle patterns were assessed within these two regions by calculating the SNR as well as the axial and lateral FWHM of the 2D ACF. In the carotid image, the CNN trained with the CS results in an SNR of 1.432 and a lateral FWHM of 493.63 μm that approximate the target values (SNR 1.261 and lateral FWHM 542.29 μm) more accurately than when trained with the VS (SNR 1.436 and lateral FWHM 474.97 μm). However, the axial FWHM of the 2D ACF with the CS is 240.23 μm, being slightly lower than that obtained with the VS (242.43 μm), with the target value of 254.15 μm. In the back image, with the BS, both axial and lateral FWHM are larger than with the VS, with the former being closer to the target. In both images, the contrasts measured in the areas delimited in magenta and blue show improvement towards the target values of −21.90 dB and −15.83 dB when training with the region-based splits, with measured contrasts of −21.15 dB in the magenta area and −16.08 dB in the blue area. In comparison, training with the CNN using the VS resulted in contrasts of −20.16 dB in the magenta area and −16.49 dB in the blue area.

To evaluate and compare the CNN’s performance, we calculated PSNR, SSIM, and KL divergence on the carotid and back test sets, as well as on the VS carotid and back test subsets. As expected, the results of the CNN trained with the CS on the carotid test set closely align with those of the VS carotid test subset, since the latter is a subset of the former. When analyzing the performance of the network trained with the region-based splits compared to the network trained with the VS, we observe that the PSNR and SSIM are lower. On the VS carotid test subset, when trained with the CS, the PSNR and SSIM are 19.441 ± 0.875 dB and 0.275 ± 0.020, respectively. However, when trained with the VS, these metrics increase to 20.402 ± 0.307 dB and 0.300 ± 0.023, respectively. This trend is consistent for the BS as well, where the PSNR and SSIM on the VS back test subset, when trained with the BS, are 19.737 ± 0.508 dB and 0.254 ± 0.033, respectively. Nonetheless, when trained with the VS they improve to 20.284 ± 0.284 dB and 0.270 ± 0.044, respectively. In both subsets, the KL divergence is marginally higher and the echogenicity distributions are slightly shifted towards lower values when training with the region-based splits instead of the VS.

The degradation of these metrics with CS and BS suggests that considering similar (i.e., from the same body region) images both in the training and testing sets enhances the final performance. However, when evaluated against the target images, the CNN trained with the CS or the BS substantially improves all metrics compared to the ones obtained with the input images. This demonstrates that the diversity of our dataset enables the network to effectively generalize to unfamiliar body regions.

### 4.3. Comparison with State-of-the-Art Methods

Evaluating the performance of different methods aiming to enhance the image quality of ultrafast US images is challenging due to the lack of consistency in the datasets and metrics used across the different studies.

Several studies aim at improving the image quality of single PW acquisitions [3,4,5,6,7,8]. Nevertheless, not all of these studies provide quantitative results on in vivo images. For instance, Perdios et al. [3,4] primarily relied on simulated data and conducted limited testing on in vivo images, without reporting any specific metrics and resorting to visual comparisons as their in vivo assessment method. On the other hand, Zhang et al. [6,7], who used self-supervised learning, mainly measured contrast-to-noise ratios to evaluate the quality of their method on in vivo images.

Zhou et al. [5] trained a GAN with an in vivo dataset of 360 image pairs, each containing focused acquisitions as target images. They assessed their results with the PSNR and SSIM. The mean PSNR improved by 17% (from 16.74 ± 1.84 dB to 19.52 ± 1.33 dB) and the SSIM improved by 77% (from 0.22 ± 0.05 to 0.39 ± 0.08), when comparing the metrics between predicted images and designated targets with respect to the values derived from the input images and the same targets.

Another study that reported the PSNR is the one by Lu et al. [8]. In this study, a CNN was trained using as input the RF images corresponding to single unfocused acquisitions and as target the IQ data resulting from compounding with three and five PWs. Their approach demonstrated a 17% improvement in mean PSNR with the three PWs target images (from 15.8 ± 1.40 dB to 18.46 ± 2.29 dB) and an 11% improvement with the five PWs target images (from 14.6 ± 1.40 dB to 16.21 ± 1.67 dB).

Remarkably, our proposed method, particularly when employing the VS, yielded higher PSNR and SSIM improvements, increasing the PSNR mean by 23% (from 16.466 ± 0.801 dB to 20.292 ± 0.307 dB) and the SSIM mean by 159% (from 0.105 ± 0.060 to 0.272 ± 0.040).

### 4.4. Computational Efficiency

The inference time of our CNN on our NVIDIA Tesla V100 GPU is, on average, less than 0.025 s. This fast inference speed is particularly valuable. Furthermore, it is worth noting that our CNN model is relatively compact in terms of its size, with a total of 4,712,609 trainable parameters.

### 4.5. Limitations and Future Work

Despite the promising results, our approach has two main limitations that need to be addressed. These limitations arise from training the CNN exclusively using in vivo data. Firstly, SLs and GLs artifacts, although highly reduced compared to the single PW case, still appear in the CPWC target images. More importantly, the PW compounding assumes that the region of interest remains stationary. Nevertheless, motion can occur between PWs acquisitions, reducing coherence between acquired signals and introducing motion artifacts in the target images. Both phenomena limit the quality of the target images, restricting the overall quality improvement that the CNN can achieve. Therefore, whereas our network successfully reduces artifacts, complete removal remains challenging. Secondly, part of our dataset consists of data acquired from body parts with a shallow depth, where deep regions contain only noise. In addition, our echogenicity values follow a Gaussian-shaped distribution, containing only a few samples for very low or very high echogenicities. Consequently, the network encounters challenges in learning from the extreme echogenicity values.

In contrast, these limitations were not present when using simulated data, as shown in [4]. Firstly, some of their target images were obtained after oversampling the transducer aperture, resulting in images with reduced GLs and higher-quality target images compared to ours. Secondly, their dataset was simulated with phantoms containing random ellipsoidal inclusions of uniformly distributed mean echogenicity in the range of −50 dB and +30 dB with respect to the background, resulting in a wider range of echogenicities with a more uniform distribution. Therefore, all echogenicities were better represented in their simulated dataset.

To tackle these constraints, future studies could explore using transfer learning from simulated to in vivo data. This could help the network to generalize from simulated to in vivo data, leading to enhanced image quality and a reduction of the number of in vivo acquisitions required to train the network.

## 5. Conclusions

Ultrafast ultrasound achieves high frame rates, but at the expense of image quality. Training a CNN on a large dataset of simulated images has been previously proposed to enhance image quality. However, the domain shift between in vivo and simulated images hindered CNN performances in practice.

To overcome this challenge, we developed a deep-learning-based method for enhancing RF images acquired with single unfocused acquisitions. This method was trained and tested on a large in vivo dataset using multiple dataset splits. To further enhance the performance of the method, we introduced a novel loss function named KLD-MSLAE. This loss outperforms MSLAE and accounts both for the high dynamic range of RF images and the echogenicity’s distribution.

Our approach yielded a substantial enhancement in image contrast and highly reduced artifacts in single unfocused in vivo acquisitions acquired in different body parts. The CNN resulted in higher PSNR and SSIM between the output and target images. Further enhancement in image quality was achieved through the adoption of the KLD-MSLAE loss, resulting in a contrast and echogenicity distribution similar to the target images. Nevertheless, the image quality enhancement was not observed when applied to the in vitro image.

We examined the CNN ability to adapt to unknown body regions, emphasizing the benefits of including similar images to the training and testing sets. The large and diverse dataset facilitated the network generalization, even when the training set did not include images acquired on the tested body region. The artifacts removal and image enhancement was observed with all dataset splits, suggesting that the dataset’s size and diversity are substantial enough to provide robust and reliable results.

Although our method faces limitations related to the limited quality of target images and the distribution of values within the dataset, it has demonstrated significant potential for reducing artifacts of ultrafast ultrasound images, which could potentially lead to more accurate analysis of tissue and flow dynamics. This work contributes to the ongoing efforts to enhance the quality of ultrafast ultrasound in vivo images. The potential impact extends to improving elastography or minimizing the number of acquisitions in portable ultrasound imaging.

## Figures and Tables

**Table 1:** Number of images and mean and standard deviation of the echogenicity values of the dataset.

	Number of Images	Echogenicity (dB)	
		1 PW	87 PWs
** Dataset**	20,000	4.65 ± 9.93	−3.83 ± 12.28
Abdomen	6599	5.43 ± 9.38	−3.08 ± 11.45
Carotids	3294	2.99 ± 10.25	−5.77 ± 13.35
Breast	3291	4.28 ± 10.50	−4.64 ± 12.96
Lower limbs	2616	6.34 ± 9.57	−0.87 ± 11.40
Upper limbs	2110	3.99 ± 10.78	−3.93 ± 13.03
Back	2090	3.93 ± 9.08	−5.43 ± 11.27

PW: plane wave.

**Table 2:** Imaging configuration and acquisitions’ parameters.

Parameter	Value
Linear array transducer	GE 9L-D
Center frequency	5.3 MHz
Bandwidth (at −6 dB)	75%
Aperture	43.93 mm
Element number	192
Pitch	230 μm
Element width ^1^	207 μm
Element height	6 mm
Elevation focus	28 mm
Transmit frequency	5.208 MHz
Excitation cycles	1
Sampling frequency	20.833 MHz
Number of compounded acquisitions	87
Steering angle spacing	0.38${}^{\circ }$
Pulse repetition frequency	9 kHz
Peak-to-peak voltage	40 V

^1^ Estimated value.

**Table 3:** Dataset splits and number of images.

	Training Set	Validation Set	Testing Set
** Volunteer-based split (VS)**	16,077	1826	2097
Carotid acquisitions	1836	594	600
Back acquisitions	1548	0	542
** Carotid split (CS)**	15,035	1671	3294
** Back split (BS)**	16,119	1791	2090

**Table 4:** Evaluation metrics computed on the highlighted areas of two in vivo acquisitions, with each color representing a distinct region in Figure 2.

	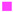 C (dB)	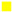 SNR	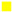 ${\mathrm{FWHM}}_{{\mathrm{ACF}}_{A}}$ (μm)	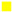 ${\mathrm{FWHM}}_{{\mathrm{ACF}}_{L}}$ (μm)	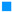 C (dB)	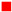 SNR	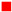 ${\mathrm{FWHM}}_{{\mathrm{ACF}}_{A}}$ (μm)	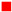 ${\mathrm{FWHM}}_{{\mathrm{ACF}}_{L}}$ (μm)
Target	−21.90	1.261	254.15	542.29	−15.83	0.838	302.12	580.19
Input	−15.28	1.451	446.78	1120.27	−11.95	1.134	260.01	222.95
KLD−MSLAE	−20.16	1.436	242.43	474.97	−16.49	0.789	296.63	764.88
MSLAE	−23.74	1.377	248.29	524.63	−18.88	0.654	337.65	1248.30

A: axial; ACF: autocovariance function; C: contrast; FWHM: full width at half maximum; KLD: Kullback–Leibler divergence; L: lateral; MSLAE: mean signed logarithmic absolute error; SNR: signal-to-noise ratio.

**Table 5:** Evaluation metrics computed on the in vivo VS test set.

	PSNR (dB)	SSIM	KL Divergence	Echogenicity (dB)
Target	-	-	-	−4.18 ± 11.64
Input	16.466 ± 0.801	0.105 ± 0.060	0.303 ± 0.090	4.48 ± 9.44
KLD-MSLAE	20.292 ± 0.307	0.272 ± 0.040	0.015 ± 0.015	−5.41 ± 11.25
MSLAE	16.196 ± 1.008	0.179 ± 0.036	0.258 ± 0.092	−13.65 ± 13.16

KL: Kullback–Leibler; PSNR: peak signal-to-noise ratio; SSIM: structural similarity index measure.

**Table 6:** Evaluation metrics computed on the in vitro acquisition. The contrasts have been computed on three inclusions, highlighted in magenta, green and blue in Figure 4. The speckle patterns have been evaluated in three areas indicated in yellow in Figure 4.

	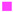 C (dB)	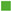 C (dB)	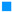 C (dB)	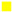 SNR	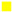 ${\mathrm{FWHM}}_{{\mathrm{ACF}}_{A}}$ (μm)	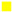 ${\mathrm{FWHM}}_{{\mathrm{ACF}}_{L}}$ (μm)
Target	−3.00	−6.27	−28.35	1.884 ± 0.039	244.38 ± 5.00	235.93 ± 8.45
Input	−3.18	−6.00	−18.33	1.911 ± 0.024	239.97 ± 4.24	239.85 ± 8.05
KLD−MSLAE	−3.60	−7.38	−20.31	1.895 ± 0.009	287.23 ± 7.63	365.10 ± 6.68
MSLAE	−4.69	−9.13	−24.04	1.658 ± 0.005	297.14 ± 6.70	405.14 ± 7.08

**Table 7:** Evaluation metrics computed on the highlighted areas in magenta and yellow of the carotid acquisition when the CNN is trained with the CS and the VS.

	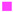 C (dB)	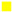 SNR	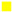 ${\mathrm{FWHM}}_{{\mathrm{ACF}}_{A}}$ (μm)	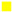 ${\mathrm{FWHM}}_{{\mathrm{ACF}}_{L}}$ (μm)
Target	−21.90	1.261	254.15	542.29
Input	−15.28	1.451	446.78	1120.27
CNN trained with the CS	−21.15	1.432	240.23	493.73
CNN trained with the VS	−20.16	1.436	242.43	474.97

**Table 8:** Evaluation metrics computed on the highlighted areas in blue and red of the back acquisition when the CNN is trained with the BS and the VS.

	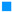 C (dB)	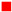 SNR	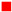 ${\mathrm{FWHM}}_{{\mathrm{ACF}}_{A}}$ (μm)	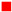 ${\mathrm{FWHM}}_{{\mathrm{ACF}}_{L}}$ (μm)
Target	−15.83	0.838	302.12	580.19
Input	−11.95	1.134	260.01	222.95
CNN trained with the BS	−16.08	0.745	306.89	851.33
CNN trained with the VS	−16.49	0.789	296.63	764.88

**Table 9:** Evaluation metrics computed on the carotid test set, the VS carotid test subset, the back test set, and the VS back test subset.

	Number of Images and Volunteers	PSNR (dB)	SSIM	KL Divergence	Echogenicity (dB)
**Carotid test set**					
Target	3294 (9 volunteers)	-	-	-	−5.77 ± 13.35
Input	3294 (9 volunteers)	16.306 ± 0.902	0.144 ± 0.060	0.291 ± 0.077	2.99 ± 10.25
CNN trained with the CS	3294 (9 volunteers)	19.159 ± 0.913	0.277 ± 0.021	0.018 ± 0.014	−8.65 ± 13.98
**VS carotid test subset**					
Target	600 (2 volunteers)	-	-	-	−4.38 ± 12.73
Input	600 (2 volunteers)	16.529 ± 0.902	0.135 ± 0.053	0.271 ± 0.073	3.99 ± 9.79
CNN trained with the CS	600 (2 volunteers)	19.441 ± 0.875	0.275 ± 0.020	0.019 ± 0.013	−7.12 ± 13.12
CNN trained with the VS	600 (2 volunteers)	20.402 ± 0.307	0.300 ± 0.023	0.018 ± 0.012	−5.94 ± 11.92
**Back test set**					
Target	2090 (9 volunteers)	-	-	-	−5.43 ± 11.27
Input	2090 (9 volunteers)	16.096 ± 0.828	0.071 ± 0.058	0.367 ± 0.112	3.93 ± 9.08
CNN trained with the BS	2090 (9 volunteers)	19.771 ± 0.517	0.259 ± 0.032	0.016 ± 0.014	−7.15 ± 11.57
**VS back test subset**					
Target	542 (2 volunteers)	-	-	-	−5.44 ± 10.98
Input	542 (2 volunteers)	16.091 ± 0.791	0.067 ± 0.054	0.372 ± 0.100	3.93 ± 8.92
CNN trained with the BS	542 (2 volunteers)	19.737 ± 0.508	0.254 ± 0.033	0.017 ± 0.012	−7.20 ± 11.32
CNN trained with the VS	542 (2 volunteers)	20.284 ± 0.284	0.270 ± 0.044	0.014 ± 0.010	−6.25 ± 10.52

## Data Availability

The data presented in this study will be made openly available.

## Abbreviations

The following abbreviations are used in this manuscript:

ACF	Autocovariance function
BMUS	British Medical Ultrasound Society
BS	Back split
C	Contrast
CNN	Convolutional neural network
CPWC	Coherent plane wave compounding
CS	Carotid split
GAN	Generative adversarial network
DW	Diverging wave
FDA	Food Drug Administration
FWHM	Full width at half maximum
GLs	Grating lobes
IQ	Phase and quadrature
ISPTA	Intensity spatial peak temporal average
KL	Kullback–Leibler
KLD	Kullback–Leibler divergence
MSLAE	Mean signed logarithmic absolute error
PW	Plane wave
PSNR	Peak signal-to-noise ratio
RF	Radio frequency
SELU	Scaled exponential linear unit
SLs	Side lobes
SNR	Signal-to-noise ratio
SSIM	Structural similarity index
US	Ultrasound
VS	Volunteer-based split
